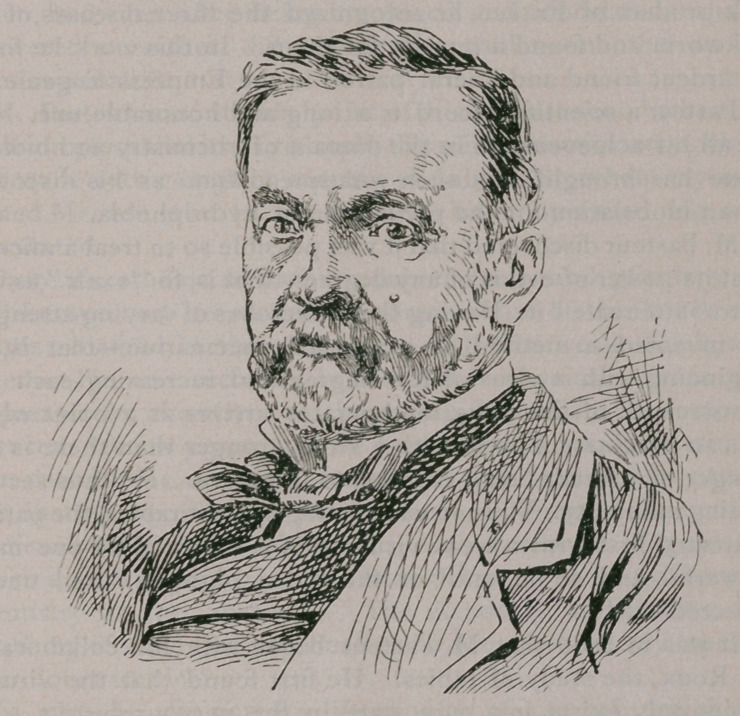# Pasteur

**Published:** 1895-11

**Authors:** 


### OBITUARY.

### PASTEUR.

M. LOUIS PASTEUR, the celebrated savant, died on September 28th. Pasteur left a name which will never be forgotten in the annals of chemistry, biology, and veterinary medicine. A chemist of repute, he utilized his knowledge of chemistry to extend it into the arena of general science; a biologist of learning, he extended his studies in a manner which could almost be called intuition, to a practical application which has been a boon to the industry of animal life. His life-work opened a field of practical investigation in the contagious and infectious diseases (comparative medicine), which brought the veterinarian into close sympathy and accord with other scientists, as the work of no man had ever done before.

Apart from his scientific labors, the life of Louis Pasteur was a very simple one. His father, a tanner, had served as a soldier under Napoleon, married and settled at Dole, where Louis was born, December 27, 1822. Two years later his parents moved to Arbois, having purchased a tannery in that town. Louis was sent to the local school, and then to Besancon College preparatory to entering the École Normale at Paris. At the École, and also at the Sorbonne, he devoted himself passionately to the study of chemistry, until he perfected himself in a knowledge that he has since exercised for the benefit of the entire human race.

So deep indeed was his devotion to the study of chemistry that upon the morning of his marriage—he was then assistant professor of chemistry at the Strasburg University—he had to be fetched from the laboratory, an incident that Mme. Pasteur has frequently smiled over when speaking of the happiness which has always attended their married life.

Pasteur's first discoveries were in regard to the fermentation of beers and wines which led to the saving of fortunes to those interested in these industries. When disease was ruining the silk product of France he recognized the three diseases of the silkworm and found a remedy for them. In this work he found an ardent friend and liberal patron in the Empress Eugenie.

Pasteur's scientific record is a long and honorable one. But of all his achievements in the domain of chemistry and biology none has brought him such widespread fame as his discovery of an inoculation for the prevention of hydrophobia.

M. Pasteur discovered that it was possible so to treat a microbe that its power of evil is of any degree—that is, to "exalt" as well as to "attenuate" it. Having these microbes of varying strengths he invented a method of graduated vaccination—that is, by beginning with a virus of low degree and increasing each day the strength of the virus, an operator arrives at a point where he can vaccinate a body with a virus stronger than there is any danger of its ever being exposed to in nature. He thus secures lasting immunity. Thus, in vaccinating against rabies, the patient is treated first with a weak virus; this is followed by one more powerful, and so on, until at the end a highly exalted one is injected safely.

It was in 1880 that M. Pasteur began with his collaborator, M. Roux, the study of rabies. He first found that the virus of rabies only exists in a pure state in the nervous system. He then found that by placing the virus directly on the surface of the brain of a dog under experiment the uncertainty of the period of incubation was done away with. Soon, after a series of inoculations from rabbit to rabbit, these animals when inoculated in the brain died from rabies after a fixed period of incubation of seven days. M. Pasteur had therefore in his possession a pure virus, with a regular and constant action, and was in a position to take the last step in the subject. As he could not act directly on the microbe of rabies, he undertook to attenuate it in its abode of predilection, the nervous tissues. He therefore took the spine of a dead rabbit and set it to dry in a bottle with two tubes containing dry air. Each day the tissue and virus lost a little of its strength, which was reduced to a minimum on the fifteenth day.

Once in possession of a scale of virulence going from the deadly nervous tissue in a fresh condition to the attenuated tissue two weeks old, he began his vaccinating experiments with dogs before and after infection by inoculating fragments of tissue mixed with sterilized water and of increasing virulence. It was found that dogs treated in this way, even after being bitten, and even after being trephined and inoculated, usually recovered, or, rather, did not develop rabies—in other words, were vaccinated.

M. Pasteur was at this point of his experiments when an occasion presented itself to apply them in a practical way, which was done with complete success in July, 1885, when a youth, named Meister, who had been badly bitten by a mad dog, was inoculated.

Pasteur's studies of anthrax, tuberculosis, and glanders are well known, and he carried the lion's share of the honor and results obtained in these diseases, with Chauveau, Koch, and others. He was successively assistant in physical science and preparator in chemistry at the Ecole Normale, and doctor of sciences A few years later he became professor in Strasburg, then dean of the scientific faculty of Lille. From this city he was, in 1857, recalled to Paris to become director of scientific studies at the Ecole Normale; then professor of geology, physics, and chemistry at the Beaux Arts; finally professor of chemistry at the Sorbonne. The Academy of Sciences, the Academy of Medicine, and, finally, the French Academy, opened their doors to him.

In 1874 he was voted a life annuity of 12,000f., which the following year was increased by 6,000f. He was made Grand Officer of the Legion of Honor in 1878. From the entire world he has received the most flattering and enviable honors and distinctions. On December 27, 1892, on the motion of the medical and surgical section of the Academy of Sciences, the seventieth anniversary of his birth was celebrated in the old Sorbonne. The President of the Republic, the Ministers, the members of Parliament, the diplomatic body, the scientific societies of France, and delegates from the universities and colleges of the whole world came and presented to Pasteur the tribute of their enthusiastic admiration.

Only once did Pasteur issue from his working retreat. This was in 1871, during the bombardment of Paris by the Germans. Pasteur wrote to the dean of the University of Bonn to request him to erase his name from the list of honorary doctors of the university "as a mark of the indignation felt by a French savant for the barbarism and hypocrisy which to satisfy criminal pride persist in the massacre of two great nations."

The funeral ceremonies of M. Pasteur took place at the Cathedral of Notre-Dame, Paris. The final interment is in the garden of the Pasteur Institute.

## Figures and Tables

**Figure f1:**